# Quantitative analysis of bone marrow fibrosis highlights heterogeneity in myelofibrosis and augments histological assessment: An Insight from a phase II clinical study of zinpentraxin alfa

**DOI:** 10.1002/hem3.105

**Published:** 2024-06-13

**Authors:** Hosuk Ryou, Korsuk Sirinukunwattana, Ruby Wood, Alan Aberdeen, Jens Rittscher, Olga K. Weinberg, Robert Hasserjian, Olga Pozdnyakova, Frank Peale, Brian Higgins, Pontus Lundberg, Kerstin Trunzer, Claire N. Harrison, Daniel Royston

**Affiliations:** ^1^ Nuffield Department of Medicine University of Oxford Oxford UK; ^2^ Ground Truth Labs, Ltd. Oxford UK; ^3^ Institute of Biomedical Engineering (IBME), Department of Engineering Science University of Oxford Oxford UK; ^4^ Big Data Institute/Li Ka Shing Centre for Health Information and Discovery University of Oxford Oxford UK; ^5^ Oxford NIHR Biomedical Research Centre Oxford University Hospitals NHS Foundation Trust Oxford UK; ^6^ Ludwig Institute for Cancer Research University of Oxford Oxford UK; ^7^ Department of Pathology University of Texas Southwestern Medical Center Dallas Texas USA; ^8^ Massachusetts General Hospital Harvard Medical School Boston Massachusetts USA; ^9^ Department of Pathology and Laboratory Medicine University of Pennsylvania Philadelphia Pennsylvania USA; ^10^ Genentech, Inc. South San Francisco California USA; ^11^ F. Hoffmann‐La Roche, Ltd. Basel Switzerland; ^12^ Guy's & St Thomas' NHS Foundation Trust London UK; ^13^ Nuffield Division of Clinical Laboratory Sciences (NDCLS), Radcliffe Department of Medicine University of Oxford Oxford UK

Accurate assessment of bone marrow fibrosis is central to the diagnosis and assessment of patients with myeloproliferative neoplasms (MPNs).^1^, ^2^, ^3^ However, European consensus criteria for fibrosis are subjective, only semiquantitative, and cannot fully capture sample fibrosis heterogeneity.^4^, ^5^, ^6^ In response, we have recently demonstrated the potential of machine learning to improve the detection and quantitation of marrow fibrosis in MPN using routinely prepared bone marrow trephine (BMT) samples.^7^ Such approaches can support accurate MPN classification/risk stratification and provide quantitative analysis of fibrosis heterogeneity, with the potential to support clinical trial teams in the evaluation of current and novel antifibrotic therapies.^6^ Here, we report evidence of such utility in the context of stage 2 of a phase II study of zinpentraxin alfa in patients diagnosed with primary or secondary myelofibrosis (MF) [ClinicalTrials.gov identifier: NCT01981850]. The primary trial endpoint was bone marrow response (≥1 grade reduction from baseline fibrosis at any timepoint). Secondary endpoints included effects on disease‐related anemia, thrombocytopenia, and constitutional symptoms.

Zinpentraxin alfa (ZPN; previously PRM‐151) is a recombinant form of human pentraxin‐2 (PTX2; also known as serum amyloid P component or SAP), a circulating endogenous regulator of the inflammatory response to tissue damage and a natural inhibitor of fibrosis.^8^, ^9^, ^10^ In the open‐label stage 1 of this phase 2 study, ZPN showed evidence of clinical activity and tolerable safety as monotherapy or in combination with ruxolitinib in patients with primary MF, post‐polycythemia vera (PV) MF, or post‐essential thrombocythemia (ET) MF.^11^ A subsequent randomized dose‐ranging study (stage 2) evaluated the efficacy and safety of three different doses of ZPN as monotherapy in patients with IPSS intermediate‐1, intermediate‐2, and high‐risk primary MF, post‐PV MF, or post‐ET MF who were anemic or thrombocytopenic and ineligible for, intolerant of, or had an inadequate prior response to ruxolitinib.^12^ Patients were randomized to receive 0.3, 3.0, or 10.0 mg/kg ZPN on Days 1, 3, and 5 of cycle 1 and every 4 weeks thereafter for up to nine cycles. Reticulin‐stained BMTs from three timepoints (screening, cycle 4 [C4D1], and cycle 9 [C9D29]) were analyzed for a subset of patients enrolled in the stage 2 study for whom digital scanned images were available at all three timepoints (50/97) (Figure 1A,B). Prior manual assessment of marrow fibrosis had been performed as part of a blinded, independent central review by three expert hematopathologists. Quantitative assessment of fibrosis using Continuous Indexing of Fibrosis (CIF) was performed by automated analyses as previously described.^7^ Briefly, CIF analysis employs a ranking convolutional neural network (CNN) trained on images of reticulin‐stained BMT slides to score image tiles for fibrosis severity. These tiles cover the analyzable marrow tissue and are used to generate fibrosis severity maps with output image scores (CIF scores) normalized between 0 and 1. Three sets of features relating to analyzed tiles are extracted from each sample: average tile CIF score, tile score distribution, and heterogeneity of CIF score. Visualization of these outputs into two‐dimensional space is performed using principal component analysis (PCA) (Figure 1C).

**Figure 1:**
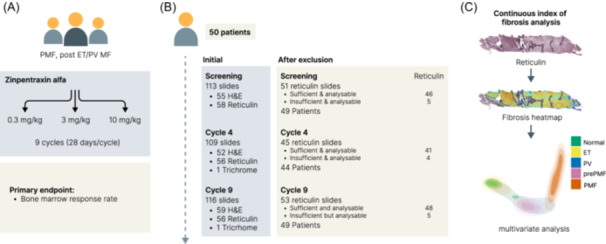
Overview of study design, quality control pipeline, and bone marrow trephine (BMT) sample analysis. (A) Patients with primary or secondary myelofibrosis (MF), previously treated with or ineligible for ruxolitinib, were randomized in a 1:1:1 ratio to receive Zinpentraxin alfa at doses of 0.3, 3, and 10 mg/kg. A 4‐week screening period was followed by 9 (4 weeks) cycles. Primary endpoint was bone marrow response rate defined as a reduction of at least one MF grade from the screening at any time. (B) Digitized images of reticulin‐stained BMTs corresponding to screening, cycle 4, and cycle 9 were available for 50 trial participants, although slides were not available for some participants at one or more time points following exclusion for quality control. All images were manually reviewed and excluded if their quality was deemed inadequate or insufficient for conventional (manual) assessment. In total, reticulin slides for all timepoints were available for 42 patients. (C) Our Continuous Indexing of Fibrosis (CIF) algorithm uses small regions (tiles) to generate a normalized score representing fibrosis severity ranging from 0 (lowest) to 1 (highest). Resulting CIF scores are visualized as a heatmap, capturing fibrosis distribution and heterogeneity across the BMT. Multivariate features extracted from this heatmap enable indexing and comparison of individual trephine slides with samples from our published reference cohort [7].

A total of 142/157 (90.4%) BMT samples obtained from 50 patients at three timepoints were evaluable. Overall, there was a moderate correlation between the average sample CIF score and the manually assigned fibrosis grade for all samples (Spearman's rho = 0.39) (Figure 2A). However, there was a marked overlap in the distribution of CIF scores across fibrosis grades, most notably for samples assigned to grades MF‐2 and MF‐3. Approximately, 38% (*n* = 16) of MF‐2 samples fell within the interquartile range of CIF distribution observed in MF‐3, and around 48% of MF‐3 (*n* = 45) samples fell within the interquartile range observed in MF‐2. This result is in keeping with the recognized challenge of accurately distinguishing between these MF grade categories, although both are consistent with a diagnosis of overt myelofibrosis. Notably, several samples manually assessed as MF‐2 had average CIF scores similar to or lower than those graded as MF‐0 or MF‐1. On review, we suspected this may reflect sample fibrosis heterogeneity; some samples with low average fibrosis (low average sample CIF score) were correctly classified as MF‐2 on the basis that ≥30% of the tissue contained more severe fibrosis (high regional CIF score). To investigate this further, we compared the ZPN trial samples taken at screening with an independent cohort of newly diagnosed and untreated MPNs in which PCA was used to combine average tile CIF score, tile score distribution, and heterogeneity of CIF score (Figure 2D). Plotting the ZPN screening samples onto this PCA of MPN “disease space” revealed that while most samples demonstrated such combined fibrosis features typical of primary or secondary myelofibrosis, several displayed features more typically seen in ET, pre‐PMF, or PV.

**Figure 2:**
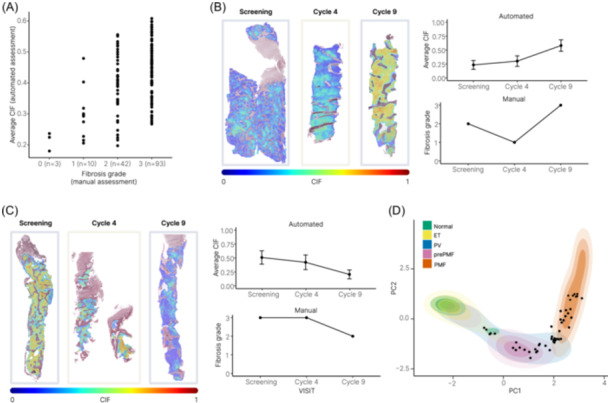
Comparison of manual bone marrow trephine (BMT) fibrosis grading and quantitative Continuous Indexing of Fibrosis (CIF) analysis. (A) Concordance of manual fibrosis grading (European Consensus) and the automated average sample CIF scores revealed a moderate correlation (Spearman's rho = 0.39). Each point represents a single slide across all trial timepoints (*n* = 148). Examples of patients with fibrotic progression (B) and improvement (C) across C4 and C9 with a comparison of CIF analysis and manual evaluation. The error bars represent one standard deviation of tile‐level CIF scores per sample. CIF “heatmaps” capture CIF scores from 0 to 1, with 0 (blue) representing no fibrosis and 1 (red) representing maximal fibrosis. (D) Visualization of screening samples [black dots] (*n* = 49) with reference to fibrosis “disease space” from a previously analyzed cohort of treatment naïve MPN samples (*n* = 107).

Having identified marked variation in both the fibrosis features at screening and average CIF scores of manually assigned MF grades for all samples, we assessed changes in fibrosis from screening to C4 and C9. This revealed an improvement in the average CIF score in 16 of 42 patients (38%) (Figure 3A). Notably, improvements in average CIF score by C9 appeared to be most marked in patients with higher CIF scores at screening, although no obvious ZPN dose‐dependent effect was observed. The overall improvement in CIF score was similar to that of manually assessed fibrosis in which 15 of 41 patients (37%) had an improvement of at least one MF grade at either C4 or C9. However, there was notable discordance between manual and quantitative CIF fibrosis assessment for individual cases (Figure 2B,C), with only 6 of 41 cases (15%) demonstrating both an improvement in CIF score and manual MF grade.

**Figure 3:**
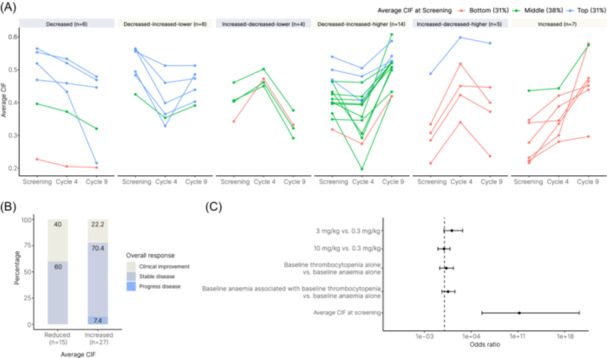
Monitoring of bone marrow trephine (BMT) Continuous Indexing of Fibrosis (CIF) scores across study timepoints and comparison to trial endpoints. (A) Evolution of CIF assessment at screening, cycle 4, and cycle 9. Each line represents a single patient (*n* = 42), with each patient stratified into six groups based on the pattern of fibrosis evolution. Patients are subdivided into three subgroups (top: blue; middle: green; bottom: red) based on the average CIF score at screening. In the top, middle, and bottom subgroups, 92% (12/13), 94% (15/16), and 23% (3/13), respectively, of patients showed improvement in fibrosis by cycle 9. (B) Stratification of patients by change in average CIF score at cycle 9 and overall response to treatment by modified IWG‐MRT criteria. (C) Logistic regression analysis on CIF score reduction. The response variable is the reduction in average CIF score at any timepoint compared to screening. Explanatory variables include treatment groups, patients' baseline hematologic status (a randomized variable), and the average CIF score at screening. The average CIF score at screening time was significantly associated with a reduction in CIF score (Wald's test *p* < 0.01).

Next, we sought to correlate changes in CIF score with the secondary trial endpoints. We observed no significant association between a change in average sample CIF score and changes in disease‐related anemia, thrombocytopenia, or constitutional symptoms (data not shown). However, we observed a trend toward an association between improving CIF score and best overall response as per modified International Working Group‐Myeloproliferative Neoplasms Research and Treatment (IWG‐MRT) criteria, with marrows from patients experiencing clinical improvement more likely to have a corresponding improvement in CIF score between screening and C9 (Figure 3B). Finally, logistic regression analysis was used to estimate the association of the treatment group, baseline anemia, or thrombocytopenia and average CIF score at screening with the reduction in average CIF score. This revealed that a higher average CIF score at screening was significantly associated with CIF score reduction for the 42 patients for whom samples were available for all three trial timepoints (Wald's test *p* < 0.01) (Figure 3C).

Our analysis is the first to demonstrate the utility of AI‐driven quantitative fibrosis analysis in a multicenter clinical trial of patients with myelofibrosis. Although CIF‐based analysis is not designed to specify an MF grade, it provides an objective measure of fibrosis severity and heterogeneity within BMTs, which is beyond conventional manual grading criteria. Moreover, it enables objective comparison across sequential samples from individual patients and allows accurate comparison within trial cohorts. Our results raise important concerns over the subjectivity of conventional fibrosis assessment in myelofibrosis, with marked overlap in CIF scores seen between and within manually assigned MF grades, and poor concordance between manually assessed and CIF‐determined fibrosis improvement. Unexpectedly, there was a marked variation in average CIF score at screening in a trial recruiting patients with high‐risk primary or secondary MF, supported by our demonstration of striking cohort heterogeneity when compared to a separate cohort of MPN. Indeed, 39% (19/49) of the screening samples analyzed in this study demonstrated fibrotic features (average severity and heterogeneity) more typical of MPNs other than primary or secondary myelofibrosis (i.e., ET, PV, and pre‐PMF). However, it should be noted that most patients recruited to this trial had high‐risk disease (39/50 with IPSS Int‐2/high risk) and 39/50 patients had received prior JAK2 inhibition. By contrast, our previously analyzed cohort of MPN included only newly diagnosed patients with no significant pretreatment. It remains unclear to what extent the inclusion of MPN patient samples with longstanding disease and/or significant pretreatment will influence our existing description of bone marrow fibrosis state in ongoing studies. Notwithstanding this caveat, our analysis suggests that variation in manual fibrosis assessment could adversely influence the accuracy and consistency of trials aiming to evaluate therapeutics targeting MF, and alternative methods for quantifying and defining fibrosis changes following therapy are indicated. This is particularly important given recent work questioning the role of marrow fibrosis assessment in evaluating outcomes in JAK inhibitor‐naïve patients treated with momelotinib or ruxolitinib, particularly as the authors relied upon local fibrosis grading with no central review.^13^ Although we could not demonstrate evidence for a significant association between CIF score improvement and the secondary clinical endpoints, we had access to WSI from only 50 of the 97 recruited patients. This reflects challenges in collecting such data as part of post hoc analytical studies and highlights the value of including such analysis in the study protocols of future clinical trials looking to evaluate bone marrow morphological response. Our observation of a trend toward an association between improving CIF score and the best overall response as per IWG‐MRT criteria warrants further evaluation of quantitative fibrosis analysis as a surrogate for clinical response in MPN trials aiming to stabilize or reverse marrow fibrosis.^14^

## AUTHOR CONTRIBUTIONS

Conception and design: Daniel Royston, Kerstin Trunzer, Korsuk Sirinukunwattana, Hosuk Ryou, Alan Aberdeen & Jens Rittscher. Collection and assembly of data: Kerstin Trunzer, Frank Peale, Brian Higgins, Pontus Lundberg, Claire N. Harrison, Olga K. Weinberg, Robert Hasserjian & Olga Pozdnyakova. Data analysis and interpretation: Hosuk Ryou, Korsuk Sirinukunwattana, Ruby Wood, Alan Aberdeen & Daniel Royston. Manuscript writing: Daniel Royston, Korsuk Sirinukunwattana, Kerstin Trunzer, Pontus Lundberg & Alan Aberdeen. Final approval of manuscript: All authors.

## CONFLICT OF INTEREST STATEMENT

Korsuk Sirinukunwattana, Alan Aberdeen, and Jens Rittscher are cofounders and equity holders of Ground Truth Labs Ltd. Daniel Royston provides consulting services to Ground Truth Labs Ltd. and Johnson & Johnson. Kerstin Trunzer and Pontus Lundberg are employees of F. Hoffmann‐La Roche and have stock ownership. Brian Higgins is an employee of F. Hoffmann‐La Roche and Genentech and has stock ownership. Frank Peale is an employee of Genentech and has stock ownership. Claire N. Harrison has received consulting fees from AbbVie, AOP, BMS, Constellation Pharmaceuticals, CTI BioPharma, Galecto, GSK, Karyopharm, Keros, MorphoSys, Novartis, Promedior, and Roche; honoraria from AbbVie, BMS, GSK, and Novartis; has advisory roles for Galecto and Keros; has received support from Novartis for attending meetings; and has a leadership or fiduciary role with the European Hematology Association and MPN Voice; and is an Editor of HemaSphere. The remaining authors declare no conflict of interest.

## FUNDING

This study was supported by F. Hoffmann‐La Roche, Ltd.; Blood Cancer UK, Grant/Award Number: 23012; Cancer Research UK, Grant/Award Number: EDDPJT‐May23/100034; EPSRC‐funded Seebibyte programme (EP/M013774/1); and Ludwig Institute for Cancer Research, Oxford Branch.

## Data Availability

The data that support the findings of this study are available from the corresponding author upon reasonable request.
